# The Attitude of the Dominion Shorthorn Breeders toward the Tuberculin Test

**Published:** 1902-02

**Authors:** W. C. Edwards


### EDITORIALS.

### THE ATTITUDE OF THE DOMINION SHORTHORN BREEDERS TOWARD THE TUBERCULIN TEST.

Last year the Central Shorthorn Breeders' Association at its annual meeting in Kansas City was persuaded, as we thought then, by Canadian influence, to incorporate into a series of resolutions some irrational, illogical, and untrue statements in regard to the tuberculin test. It devolved upon us at that time to point out the inaccuracies and falsifications in these resolutions and to explain why the shorthorn breeders should discuss the tuberculosis question in a sober, temperate way.

Our explanation and protest seem to have had some effect, for this year the Central Shorthorn Breeders' Association did not repeat its foolish tactics.

But the Dominion Shorthorn Breeders have just had a meeting and they have adopted the following resolution :

"We, the Dominion Shorthorn Breeders' Association, at our annual meeting, resolve that the tuberculin test is unreliable, unnecessary, and in many cases injurious, and we urge the discontinuance of the compulsory use of the toxin by the Dominion Government Department of Agriculture."

It is stated that the resolution carried unanimously.

Three distinct statements, or charges, are made against tuberculin in this resolution. It is alleged that it is : 1, unreliable ; 2, unnecessary, and 3, that it is injurious.

This attitude toward the tuberculin test is a reflection of the opinions of some breeders in England, and may be regarded as a view imported from that country. When, however, we recall the fact that shorthorn cattle have done more than those of any other breed to spread tuberculosis among the cattle of the world, and that it is rare to find a pure bred herd that will pass the tuberculin test, we find a possible reason for the hysterical vehemence of the Dominion Shorthorn Breeders.

The tuberculosis question is a great question. It is the greatest question now up for settlement before the breeders of all countries. It can only be settled by calm, careful study by competent men working with adequate facilities. To throw stones and tell lies will not contribute to but will retard the solution of this problem. For this reason we regret the attitude of the Dominion Shorthorn Breeders. They have set themselves down as a body of men in no frame of mind to deal with a great and serious menace to the cattle interests in general and to their breed in particular.

In making this statement we wish it to be understood that we speak of the Association as a whole and not of the individuals composing it. As an individual, each Shorthorn breeder is as amenable to reason as is any other breeder, but together they do foolish things, as crowds sometimes do. Back of it all must be the fear that their herds are vulnerable, for the Hereford, Ayrshire, and Angus breeders do not do these silly things.

As to the allegations contained in the resolutions :

1. The tuberculin test has itself been tested as has no other method for diagnosing any disease of man or animals. It has been tried by government commissions and experiment stations in all civilized parts of the world. After these trials, going on for ten years, it stands as incomparably the most reliable test or means for recognizing tuberculosis in animals, and, more than this, as the most accurate means now in use for the diagnosis of any special internal disease. Is it infallible? No. What is infallible in diagnosis or treatment? Nothing.

2. As to whether the tuberculin test is necessary or not depends upon whether it is necessary to prevent traffic in tuberculous cattle. If it is necessary to prevent such traffic, no better way to do it has been devised than that afforded by the tuberculin test. Of course, the Shorthorn breeders who have tuberculous cattle to sell think that such prohibition is unnecessary and is burdensome. We will admit that it is burdensome to be refused the privilege of infecting your customer's herd, but the customers think it is necessary. So here is a perfectly natural difference of opinion.

3. The charge that the tuberculin test is injurious shows a *naiveté* among the Dominion Shorthorn Breeders that is interesting. This old charge was exploded years ago. All of the experiment stations have shown, and thousands of tests prove, that the test is harmless. But, of course, there is another side to this too. The test *is* sometimes injurious—to the pocket of the man who hopes to get a high price for a tuberculous cow.

Now, Messrs. Dominion Shorthorn Breeders, *be men.* Tell the truth. Instead of growling at the attempts your government is making to free you from this scourge, devise a better plan if you do not like the present one. There can be no doubt that a better plan will be adopted as soon as one becomes available.

Instead of grumbling at men who do not want to buy tuberculous cattle, free your own herds of disease, so that you will have none to sell. When your herds are clean you will find your objections to the tuberculin test have vanished.

You have before you an instructive and inspiring example in the work that has been done so successfully by your distinguished and public spirited fellow-breeder, Hon. W. C. Edwards.

